# Dental Caries

**Published:** 1890-02

**Authors:** Henry Sewill


ARTICLE V.

### DENTAL CARIES.

BY HENRY SEWILL, M.R.C.S. AND L.D.S., ENG.

The only remote or predisposing causes of caries of which the existence has been demonstrated, and of which the action is demonstrable, are those named in my papers, namely, inherent structural defects in enamel, vitiation of the buccal secretions, and crowding and irregularity of the teeth. The statement that enamel, through causes acting from within a tooth, can undergo a process of softening or deterioration—a kind of degeneration—rendering it less able to withstand attacks of caries, is pure hypothesis, resting on very insufficient foundation ; and it is besides entirely unnecessary, all the phenomena being accounted for without its introduction. If any one really believed that enamel were capable of physiological, and therefore of pathological action, he would never fill a simple cavity of decay. It is to be believed that a tissue so highly organized as the hypothesis in question supposes, would passively tolerate the presence of a foreign body like a stopping wedged into its substance ? One consideration like this is alone almost enough, to show the falsity of the views upon which you have asked my opinion; but there are many more equally cogent. The amount of organic matter in enamel is so minute as to be indistinguishable by the microscope, and we are justified in affirming that enamel is devoid of those tissue elements without which physiological action is impossible. An American observer states that he has stained enamel with chloride of gold, but if this observation were correct—as to which there are grave doubts—the organic matter must be in a state of almost inconceivable tenuity. You will see from my book that Mr. Charters White, one of the most distinguished living dental histologists, agrees with me that there is probably some error in the observation. But if it were true, can we imagine the passage to and fro of nutritive and effete material via the dental fibrils to the surface of the enamel; and can we imagine their assimilation and rejection by a quartz-like inert mass such as composes almost the entire bulk of enamel? And furthermore, if in some systemic states teeth were to undergo degeneration, owing to abstraction of their solid constituents through the vascular system, surely the morbid process would begin, if not always, at least very often in the surfaces nearest the vessels—in the cementum and the dentine forming the walls of the pulp cavity? Does any one allege that he has observed such a phenomenon? and can any one produce a single specimen of enamel in process of softening or disintegration, displaying any appearance not equally visible in a carious dead tooth. Indeed, with the exception of pain, the single subjective symptom of caries, all the phenomena of this malady, whether as regards appearances visible to the naked eye or disclosed by the microscope, are to be observed not only in the dead human teeth replaced in the mouth as artificial substitutes, but in blocks of ivory used for the same purpose. And the remote as well as direct causes of decay in these dead substances when worn in the mouth, are precisely the same as govern the onset of caries in living teeth —teeth with living pulps and living periosteum. Dead teeth and ivory blocks are under similar conditions neither more nor less liable to decay in the mouth than their neighbors implanted in the alveoli. Some few years ago, before the general use of vulcanite, artificial teeth were much more frequently constructed of gold plates with human teeth mounted upon them, and it was a fact of common observation—one which I was able fully to verify—that the durability of this kind of work varied much in different individuals and under changing circumstances in the same individual. Every dentist recognized that their durability depended very largely upon the quality of the teeth and blocks employed; if these were of the most solid structure they lasted much longer than if inherently weak. Everyone recognized also that their durability depended, secondly, on the healthy and personal habits of the wearer. In a mouth habitually neglected and where the frames were allowed to remain for long periods coated with decomposing debris, the dead teeth and ivory were speedily softened and destroyed; whilst on the other hand where the mouth and teeth were kept scrupulously clean the beginning of decay was proportionately less frequent and its progress in like degree less rapid. A combination of bad health with neglect, giving rise to extreme vitiation of the buccal secretions, was with certainty accompanied by destruction of the artificial teeth. In short it is amply proved, that disturbances of the general health exercise the same indirect influence upon ivory blocks worn in the mouth, as upon living teeth, and the effects are traceable onwards through the same agencies, namely, putrefaction and fermentation of organic matter attended by formation of acids and development of micro-organisms in the vicinity and on the surface of the teeth. This is what happens in the cases of pregnancy about which you write. It is only a minority of women whose teeth suffer during that period, and in these there is almost invariably present dyspepsia with local conditions such as I have just referred to.

There has probably been more nonsense written on the subject of dental caries than on any other topic of the sort, and I have no doubt that the same kind of writing will go on in the United States, so long as dental societies and dental journals refrain from holding up pseudo-scientific pretenders to the ridicule they deserve. We feel a solidarity in this country with our professional brethren across the Atlantic, and we take deep interest in all that concerns the progress of the profession in the great Republic. But beyond that, I do not think that the production in America of sham scientific dental literature, whether in the form of papers or of text books and manuals for students will affect us injuriously. Our students are not likely to go astray in these matters. They are all obliged before commencing their special studies to pass an examination in general education, and having passed that examination they are not likely to pay much attention to authors, whose writings glaringly make manifest not only their ignorance of the meaning of the scientific terms which they glibly use, but their want of acquaintance with the rules of language and grammar, without which the simplest scientific proposition cannot be clearly expressed. It was partly to expose the worthlessness of the productions of writers of this class that I composed the papers to which you so kindly refer; and I am quite satisfied to know that they have not been altogether without effect in checking an evil from which we on this side of the Atlantic have been by no means exempt.—*Dominion Dental Journal.*

